# 2,2-Diphenyl-*N*-(2,4,5-trichloro­phen­yl)acetamide

**DOI:** 10.1107/S160053681203440X

**Published:** 2012-08-08

**Authors:** Hoong-Kun Fun, Ching Kheng Quah, Prakash S. Nayak, B. Narayana, B. K. Sarojini

**Affiliations:** a X-ray Crystallography Unit, School of Physics, Universiti Sains Malaysia, 11800 USM, Penang, Malaysia; b Department of Studies in Chemistry, Mangalore University, Mangalagangotri 574 199, India; c Department of Chemistry, P.A. College of Engineering, Nadupadavu, Mangalore 574 153, India

## Abstract

The asymmetric unit of the title compound, C_20_H_14_Cl_3_NO, consists of two independent mol­ecules. In one mol­ecule, the chlorinated benzene ring forms dihedral angles of 12.00 (9) and 77.04 (9)° with the phenyl rings. The dihedral angle between the phenyl rings is 80.37 (10)°. The corresponding dihedral angles for the other mol­ecule are 26.34 (10), 62.98 (10) and 88.47 (11)°, respectively. One of the mol­ecules features an intra­molecular C—H⋯O hydrogen bond, which forms an *S*(6) ring motif. In the crystal, mol­ecules are linked by N—H⋯O hydrogen bonds into [100] chains. The chains are further linked by C—H⋯O and C—H⋯Cl hydrogen bonds into a three-dimensional network.

## Related literature

For general background to and related structures of the title compound, see: Fun *et al.* (2011*a* ▶,*b* ▶, 2012*a* ▶,*b* ▶). For the stability of the temperature controller used for the data collection, see: Cosier & Glazer (1986 ▶). For hydrogen-bond motifs, see: Bernstein *et al.* (1995 ▶).
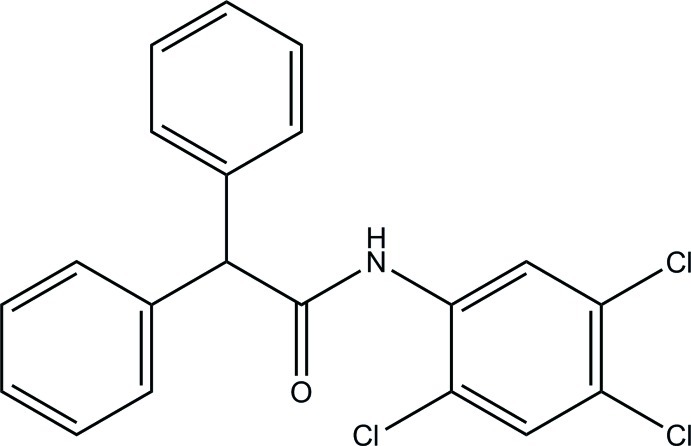


## Experimental

### 

#### Crystal data

C_20_H_14_Cl_3_NO
*M* *_r_* = 390.67
Orthorhombic, 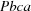

*a* = 18.6630 (16) Å
*b* = 17.1713 (15) Å
*c* = 22.5648 (19) Å
*V* = 7231.3 (11) Å^3^
*Z* = 16
Mo *K*α radiation
μ = 0.51 mm^−1^
*T* = 100 K
0.38 × 0.14 × 0.11 mm

#### Data collection

Bruker SMART APEXII DUO CCD diffractometer
Absorption correction: multi-scan (*SADABS*; Bruker, 2009 ▶) *T*_min_ = 0.830, *T*_max_ = 0.946
45180 measured reflections
10676 independent reflections
7536 reflections with *I* > 2σ(*I*)
*R*_int_ = 0.076

#### Refinement

*R*[*F* ^2^ > 2σ(*F* ^2^)] = 0.047
*wR*(*F* ^2^) = 0.114
*S* = 1.02
10676 reflections
459 parameters
H atoms treated by a mixture of independent and constrained refinement
Δρ_max_ = 0.52 e Å^−3^
Δρ_min_ = −0.49 e Å^−3^

### 

Data collection: *APEX2* (Bruker, 2009 ▶); cell refinement: *SAINT* (Bruker, 2009 ▶); data reduction: *SAINT*; program(s) used to solve structure: *SHELXTL* (Sheldrick, 2008 ▶); program(s) used to refine structure: *SHELXTL*; molecular graphics: *SHELXTL*; software used to prepare material for publication: *SHELXTL* and *PLATON* (Spek, 2009 ▶).

## Supplementary Material

Crystal structure: contains datablock(s) global, I. DOI: 10.1107/S160053681203440X/hb6924sup1.cif

Structure factors: contains datablock(s) I. DOI: 10.1107/S160053681203440X/hb6924Isup2.hkl

Supplementary material file. DOI: 10.1107/S160053681203440X/hb6924Isup3.cml

Additional supplementary materials:  crystallographic information; 3D view; checkCIF report

## Figures and Tables

**Table 1:** Hydrogen-bond geometry (Å, °)

*D*—H⋯*A*	*D*—H	H⋯*A*	*D*⋯*A*	*D*—H⋯*A*
N1*A*—H1*NA*⋯O1*B* ^i^	0.78 (2)	2.09 (2)	2.8379 (19)	161 (2)
N1*B*—H1*NB*⋯O1*A*	0.84 (2)	1.94 (2)	2.7684 (19)	168 (2)
C7*A*—H7*AA*⋯O1*B* ^i^	1.00	2.33	3.234 (2)	151
C1*B*—H1*BA*⋯O1*B*	0.95	2.48	3.116 (3)	125
C3*B*—H3*BA*⋯O1*B* ^ii^	0.95	2.42	3.368 (3)	172
C12*B*—H12*B*⋯Cl2*B* ^iii^	0.95	2.82	3.641 (3)	145
C7*B*—H7*BA*⋯O1*A*	1.00	2.46	3.341 (2)	147

Symmetry codes: (i) 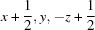; (ii) 
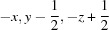
; (iii) 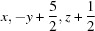.

**Fig. 1.**
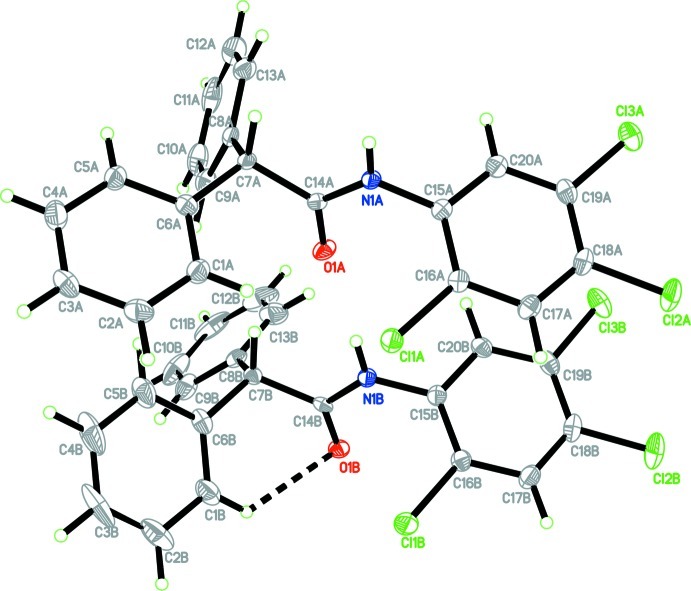
The asymmetric unit of the title compound showing 50% probability displacement ellipsoids for non-H atoms. Intramolecular hydrogen bond is shown as dashed line.

**Fig. 2.**
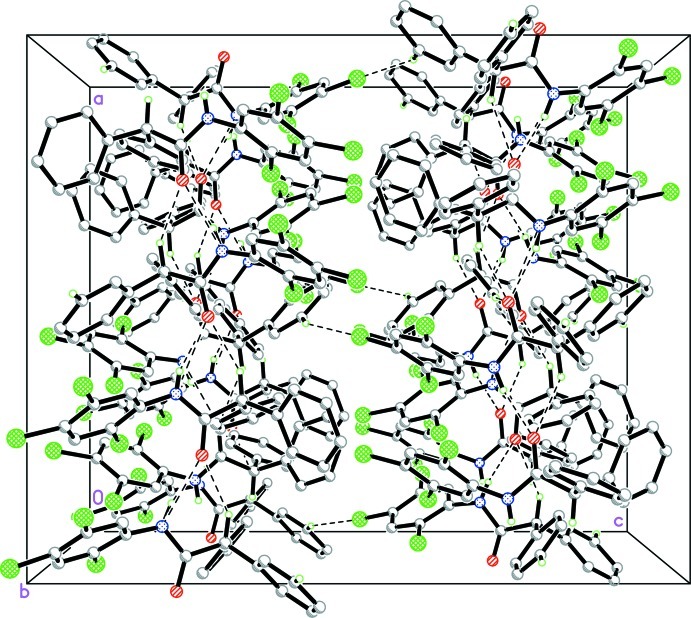
The crystal structure of the title compound, viewed along the b axis. H atoms not involved in hydrogen bonds (dashed lines) have been omitted for clarity.

## supplementary crystallographic information

### Comment

In continuation of our work on synthesis of amides (Fun *et al.*, 2011*a*, 2011*b*, 2012*a*, 2012*b*), we report herein the crystal structure of the title compound.

The asymmetric unit (Fig. 1) of the title compound consists of two independent molecules (*A* and *B*), with comparable geometries. In molecule *A*, the benzene ring (C15A-C20A) forms dihedral angles of 12.00 (9) and 77.04 (9)° with two phenyl rings (C1A-C6A and C8A-C13A). The dihedral angle between two phenyl rings is 80.37 (10)°. The corresponding dihedral angles for molecule *B* are 26.34 (10), 62.98 (10) and 88.47 (11)°, respectively. Bond lengths and angles are within normal ranges and are comparable to related structures (Fun *et al.*, 2011*a*, 2011*b*, 2012*a*, 2012*b*). The molecular structure is stabilized by intramolecular C1B–H1BA···O1B hydrogen bond, forming an S(6) ring motif (Bernstein *et al.*, 1995).

In the crystal structure, Fig. 2, molecules are linked by N1A–H1NA···O1B, N1B–H1NB···O1A, C7A–H7AA···O1B, C3B–H3BA···O1B, C12B–H12B···Cl2B and C7B–H7BA···O1A hydrogen bonds (Table 1) into a three-dimensional network.

### Experimental

Diphenylacetic acid (0.212 g, 1 mmol), 2,4,5-trichloroaniline (0.196 g, 1 mmol) and 1-ethyl-3-(3-dimethylaminopropyl)-carbodiimide hydrochloride (1.0 g, 0.01 mol) were dissolved in dichloromethane (20 ml). The mixture was stirred in the presence of triethylamine at 273 K for about 3 h. The contents were poured into 100 ml of ice-cold aqueous hydrochloric acid with stirring. The concoction was extracted thrice with dichloromethane. The organic layer was washed with saturated NaHCO_3_ solution and brine solution, dried and concentrated under reduced pressure to give the title compound (I). Colourless needles were grown from ethanol solution by the slow evaporation method (*m.p.*: 391-393K).

### Refinement

N-bound H atoms were located in a difference Fourier map and refined freely [N–H = 0.78 (2) or 0.84 (2) Å]. The remaining H atoms were positioned geometrically and refined using a riding model with C–H = 0.95 or 1.00 Å and *U*_iso_(H) = 1.2 *U*_eq_(C).

### Crystal data

**Table:**

C_20_H_14_Cl_3_NO	*F*(000) = 3200
*M**_r_* = 390.67	*D*_x_ = 1.435 Mg m^−^^3^
Orthorhombic, *P**b**c**a*	Mo *K*α radiation, λ = 0.71073 Å
Hall symbol: -P 2ac 2ab	Cell parameters from 6747 reflections
*a* = 18.6630 (16) Å	θ = 2.4–29.9°
*b* = 17.1713 (15) Å	µ = 0.51 mm^−^^1^
*c* = 22.5648 (19) Å	*T* = 100 K
*V* = 7231.3 (11) Å^3^	Needle, colourless
*Z* = 16	0.38 × 0.14 × 0.11 mm

### Data collection

**Table:**

Bruker SMART APEXII DUO CCD diffractometer	10676 independent reflections
Radiation source: fine-focus sealed tube	7536 reflections with *I* > 2σ(*I*)
Graphite monochromator	*R*_int_ = 0.076
φ and ω scans	θ_max_ = 30.2°, θ_min_ = 1.8°
Absorption correction: multi-scan (*SADABS*; Bruker, 2009)	*h* = −26→25
*T*_min_ = 0.830, *T*_max_ = 0.946	*k* = −15→24
45180 measured reflections	*l* = −28→31

### Refinement

**Table:**

Refinement on *F*^2^	Primary atom site location: structure-invariant direct methods
Least-squares matrix: full	Secondary atom site location: difference Fourier map
*R*[*F*^2^ > 2σ(*F*^2^)] = 0.047	Hydrogen site location: inferred from neighbouring sites
*wR*(*F*^2^) = 0.114	H atoms treated by a mixture of independent and constrained refinement
*S* = 1.02	*w* = 1/[σ^2^(*F*_o_^2^) + (0.0465*P*)^2^ + 2.0216*P*] where *P* = (*F*_o_^2^ + 2*F*_c_^2^)/3
10676 reflections	(Δ/σ)_max_ = 0.001
459 parameters	Δρ_max_ = 0.52 e Å^−^^3^
0 restraints	Δρ_min_ = −0.49 e Å^−^^3^

### Special details

**Table:**

Experimental. The crystal was placed in the cold stream of an Oxford Cryosystems Cobra open-flow nitrogen cryostat (Cosier & Glazer, 1986) operating at 100.0 (1) K.
Geometry. All esds (except the esd in the dihedral angle between two l.s. planes) are estimated using the full covariance matrix. The cell esds are taken into account individually in the estimation of esds in distances, angles and torsion angles; correlations between esds in cell parameters are only used when they are defined by crystal symmetry. An approximate (isotropic) treatment of cell esds is used for estimating esds involving l.s. planes.
Refinement. Refinement of F^2^ against ALL reflections. The weighted R-factor wR and goodness of fit S are based on F^2^, conventional R-factors R are based on F, with F set to zero for negative F^2^. The threshold expression of F^2^ > 2sigma(F^2^) is used only for calculating R-factors(gt) etc. and is not relevant to the choice of reflections for refinement. R-factors based on F^2^ are statistically about twice as large as those based on F, and R- factors based on ALL data will be even larger.

### Fractional atomic coordinates and isotropic or equivalent isotropic displacement parameters (Å^2^)

**Table:**

	*x*	*y*	*z*	*U*_iso_*/*U*_eq_	
Cl1A	0.25176 (2)	0.93641 (3)	0.13414 (2)	0.01907 (10)	
Cl2A	0.27575 (3)	1.19747 (3)	0.00787 (2)	0.02842 (12)	
Cl3A	0.36832 (3)	1.27469 (3)	0.11150 (2)	0.02949 (12)	
O1A	0.23516 (6)	1.01068 (8)	0.26465 (6)	0.0181 (3)	
N1A	0.34664 (8)	1.01920 (9)	0.22602 (7)	0.0137 (3)	
C1A	0.28328 (10)	0.83104 (12)	0.27665 (9)	0.0210 (4)	
H1AA	0.2713	0.8590	0.2417	0.025*	
C2A	0.27020 (11)	0.75132 (13)	0.27947 (9)	0.0246 (4)	
H2AA	0.2492	0.7256	0.2465	0.030*	
C3A	0.28742 (10)	0.70921 (12)	0.32957 (9)	0.0236 (4)	
H3AA	0.2783	0.6548	0.3314	0.028*	
C4A	0.31836 (11)	0.74771 (13)	0.37728 (9)	0.0265 (4)	
H4AA	0.3308	0.7194	0.4119	0.032*	
C5A	0.33122 (11)	0.82721 (12)	0.37473 (8)	0.0217 (4)	
H5AA	0.3523	0.8527	0.4077	0.026*	
C6A	0.31349 (9)	0.87024 (11)	0.32420 (8)	0.0150 (3)	
C7A	0.33187 (9)	0.95686 (10)	0.32258 (7)	0.0131 (3)	
H7AA	0.3852	0.9615	0.3198	0.016*	
C8A	0.30808 (9)	1.00164 (11)	0.37751 (8)	0.0149 (3)	
C9A	0.23862 (10)	0.99421 (12)	0.39993 (8)	0.0208 (4)	
H9AA	0.2064	0.9578	0.3828	0.025*	
C10A	0.21668 (12)	1.04027 (13)	0.44749 (9)	0.0281 (5)	
H10A	0.1692	1.0357	0.4623	0.034*	
C11A	0.26365 (14)	1.09258 (14)	0.47326 (9)	0.0345 (6)	
H11A	0.2483	1.1241	0.5055	0.041*	
C12A	0.33262 (14)	1.09906 (13)	0.45230 (10)	0.0323 (5)	
H12A	0.3651	1.1342	0.4706	0.039*	
C13A	0.35485 (11)	1.05418 (12)	0.40421 (9)	0.0233 (4)	
H13A	0.4023	1.0595	0.3895	0.028*	
C14A	0.29980 (9)	0.99760 (10)	0.26836 (7)	0.0129 (3)	
C15A	0.32606 (9)	1.06227 (11)	0.17496 (7)	0.0135 (3)	
C16A	0.28376 (9)	1.03068 (11)	0.12995 (8)	0.0148 (3)	
C17A	0.26736 (10)	1.07342 (12)	0.07947 (8)	0.0185 (4)	
H17A	0.2379	1.0514	0.0495	0.022*	
C18A	0.29391 (10)	1.14804 (12)	0.07292 (8)	0.0185 (4)	
C19A	0.33520 (10)	1.18112 (11)	0.11770 (8)	0.0189 (4)	
C20A	0.35102 (9)	1.13825 (11)	0.16837 (8)	0.0171 (4)	
H20A	0.3792	1.1611	0.1988	0.020*	
Cl1B	0.03020 (3)	0.94169 (3)	0.09896 (2)	0.02343 (11)	
Cl2B	0.05234 (3)	1.22599 (4)	0.00236 (2)	0.03695 (14)	
Cl3B	0.14530 (3)	1.28165 (3)	0.11307 (2)	0.02957 (12)	
O1B	−0.01287 (6)	1.01048 (8)	0.22632 (6)	0.0165 (3)	
N1B	0.10563 (8)	1.01476 (9)	0.20494 (7)	0.0149 (3)	
C1B	0.00063 (12)	0.83378 (13)	0.25576 (9)	0.0279 (5)	
H1BA	−0.0283	0.8675	0.2325	0.034*	
C2B	−0.01116 (14)	0.75387 (14)	0.25393 (11)	0.0385 (6)	
H2BA	−0.0481	0.7332	0.2296	0.046*	
C3B	0.03066 (13)	0.70482 (13)	0.28732 (13)	0.0423 (7)	
H3BA	0.0223	0.6503	0.2863	0.051*	
C4B	0.08436 (13)	0.73425 (14)	0.32218 (14)	0.0465 (7)	
H4BA	0.1135	0.7001	0.3449	0.056*	
C5B	0.09604 (11)	0.81390 (13)	0.32417 (11)	0.0333 (5)	
H5BA	0.1333	0.8340	0.3485	0.040*	
C6B	0.05443 (9)	0.86469 (11)	0.29132 (8)	0.0187 (4)	
C7B	0.06907 (9)	0.95122 (10)	0.29632 (8)	0.0144 (3)	
H7BA	0.1218	0.9576	0.3024	0.017*	
C8B	0.03170 (9)	0.99156 (11)	0.34826 (8)	0.0171 (4)	
C9B	−0.01905 (10)	0.95404 (13)	0.38322 (9)	0.0249 (4)	
H9BA	−0.0318	0.9016	0.3751	0.030*	
C10B	−0.05113 (12)	0.99360 (17)	0.43017 (9)	0.0370 (6)	
H10B	−0.0853	0.9676	0.4543	0.044*	
C11B	−0.03377 (13)	1.06999 (18)	0.44199 (10)	0.0423 (7)	
H11B	−0.0557	1.0965	0.4742	0.051*	
C12B	0.01588 (13)	1.10794 (16)	0.40664 (11)	0.0413 (6)	
H12B	0.0276	1.1608	0.4142	0.050*	
C13B	0.04849 (12)	1.06879 (13)	0.36023 (10)	0.0295 (5)	
H13B	0.0827	1.0951	0.3363	0.035*	
C14B	0.04969 (9)	0.99439 (10)	0.23948 (8)	0.0133 (3)	
C15B	0.09611 (9)	1.06242 (11)	0.15425 (8)	0.0151 (3)	
C16B	0.05996 (10)	1.03687 (11)	0.10398 (8)	0.0169 (4)	
C17B	0.04756 (10)	1.08745 (12)	0.05716 (8)	0.0220 (4)	
H17B	0.0220	1.0700	0.0233	0.026*	
C18B	0.07236 (10)	1.16331 (12)	0.05979 (8)	0.0221 (4)	
C19B	0.11177 (10)	1.18824 (11)	0.10842 (9)	0.0200 (4)	
C20B	0.12309 (9)	1.13779 (11)	0.15547 (8)	0.0178 (4)	
H20B	0.1496	1.1550	0.1889	0.021*	
H1NA	0.3879 (12)	1.0179 (13)	0.2313 (10)	0.024 (6)*	
H1NB	0.1474 (12)	1.0101 (13)	0.2186 (10)	0.022 (6)*	

### Atomic displacement parameters (Å^2^)

**Table:**

	*U*^11^	*U*^22^	*U*^33^	*U*^12^	*U*^13^	*U*^23^
Cl1A	0.0270 (2)	0.0140 (2)	0.0162 (2)	−0.00103 (17)	−0.00160 (17)	−0.00142 (16)
Cl2A	0.0418 (3)	0.0240 (3)	0.0194 (2)	0.0052 (2)	−0.0025 (2)	0.00825 (19)
Cl3A	0.0362 (3)	0.0173 (2)	0.0349 (3)	−0.0061 (2)	−0.0022 (2)	0.0071 (2)
O1A	0.0105 (6)	0.0288 (8)	0.0151 (6)	0.0016 (5)	−0.0005 (5)	0.0017 (5)
N1A	0.0081 (7)	0.0183 (8)	0.0146 (7)	0.0008 (6)	0.0000 (5)	0.0031 (6)
C1A	0.0255 (9)	0.0192 (10)	0.0183 (9)	−0.0003 (8)	−0.0026 (8)	0.0003 (7)
C2A	0.0264 (10)	0.0216 (11)	0.0257 (10)	−0.0030 (8)	−0.0025 (8)	−0.0063 (8)
C3A	0.0233 (9)	0.0151 (10)	0.0325 (11)	−0.0017 (8)	0.0054 (8)	−0.0011 (8)
C4A	0.0342 (11)	0.0205 (11)	0.0248 (11)	−0.0007 (9)	0.0000 (8)	0.0048 (8)
C5A	0.0281 (10)	0.0199 (10)	0.0172 (9)	−0.0021 (8)	−0.0035 (8)	0.0014 (8)
C6A	0.0116 (7)	0.0163 (9)	0.0169 (9)	0.0007 (7)	0.0025 (6)	−0.0007 (7)
C7A	0.0097 (7)	0.0166 (9)	0.0129 (8)	−0.0008 (6)	−0.0004 (6)	0.0017 (7)
C8A	0.0196 (8)	0.0139 (9)	0.0113 (8)	0.0011 (7)	−0.0020 (6)	0.0023 (6)
C9A	0.0233 (9)	0.0242 (11)	0.0150 (9)	0.0021 (8)	0.0021 (7)	0.0024 (8)
C10A	0.0366 (12)	0.0296 (12)	0.0182 (10)	0.0110 (10)	0.0091 (8)	0.0058 (8)
C11A	0.0682 (17)	0.0205 (12)	0.0149 (10)	0.0140 (11)	0.0021 (10)	0.0006 (8)
C12A	0.0540 (15)	0.0186 (11)	0.0242 (11)	−0.0014 (10)	−0.0103 (10)	−0.0036 (9)
C13A	0.0288 (10)	0.0192 (10)	0.0217 (10)	−0.0007 (8)	−0.0068 (8)	−0.0001 (8)
C14A	0.0118 (7)	0.0141 (9)	0.0126 (8)	−0.0006 (6)	−0.0008 (6)	−0.0012 (6)
C15A	0.0108 (7)	0.0171 (9)	0.0125 (8)	0.0032 (6)	0.0023 (6)	−0.0010 (7)
C16A	0.0156 (8)	0.0136 (9)	0.0152 (8)	0.0030 (7)	0.0021 (6)	−0.0012 (7)
C17A	0.0213 (9)	0.0199 (10)	0.0143 (9)	0.0041 (7)	−0.0002 (7)	−0.0017 (7)
C18A	0.0208 (9)	0.0199 (10)	0.0149 (9)	0.0063 (7)	0.0022 (7)	0.0036 (7)
C19A	0.0196 (9)	0.0147 (9)	0.0224 (9)	0.0006 (7)	0.0035 (7)	0.0025 (7)
C20A	0.0157 (8)	0.0192 (10)	0.0163 (9)	0.0014 (7)	0.0022 (7)	−0.0002 (7)
Cl1B	0.0310 (2)	0.0175 (2)	0.0218 (2)	−0.00232 (19)	−0.00480 (19)	−0.00196 (18)
Cl2B	0.0515 (3)	0.0325 (3)	0.0269 (3)	0.0077 (3)	0.0011 (2)	0.0155 (2)
Cl3B	0.0337 (3)	0.0162 (2)	0.0388 (3)	−0.0046 (2)	0.0087 (2)	0.0030 (2)
O1B	0.0105 (5)	0.0190 (7)	0.0202 (7)	0.0003 (5)	−0.0017 (5)	0.0006 (5)
N1B	0.0090 (7)	0.0190 (8)	0.0166 (8)	−0.0003 (6)	−0.0006 (6)	0.0040 (6)
C1B	0.0356 (11)	0.0189 (11)	0.0294 (11)	−0.0075 (9)	0.0040 (9)	−0.0025 (8)
C2B	0.0474 (14)	0.0222 (12)	0.0457 (14)	−0.0140 (11)	0.0154 (11)	−0.0112 (10)
C3B	0.0407 (13)	0.0121 (11)	0.0743 (19)	−0.0034 (10)	0.0377 (13)	−0.0022 (11)
C4B	0.0315 (12)	0.0209 (13)	0.087 (2)	0.0072 (10)	0.0160 (13)	0.0204 (13)
C5B	0.0228 (10)	0.0209 (11)	0.0562 (15)	0.0019 (9)	−0.0001 (10)	0.0132 (10)
C6B	0.0165 (8)	0.0146 (9)	0.0249 (10)	0.0015 (7)	0.0077 (7)	0.0009 (7)
C7B	0.0106 (7)	0.0141 (9)	0.0184 (9)	−0.0005 (6)	0.0000 (6)	0.0014 (7)
C8B	0.0156 (8)	0.0194 (10)	0.0165 (9)	0.0049 (7)	−0.0047 (7)	−0.0011 (7)
C9B	0.0220 (9)	0.0321 (12)	0.0207 (10)	0.0073 (8)	0.0013 (8)	0.0046 (8)
C10B	0.0299 (11)	0.0636 (19)	0.0175 (10)	0.0184 (11)	0.0025 (9)	0.0057 (11)
C11B	0.0391 (13)	0.068 (2)	0.0196 (11)	0.0304 (13)	−0.0111 (10)	−0.0171 (11)
C12B	0.0428 (14)	0.0407 (15)	0.0404 (14)	0.0151 (12)	−0.0165 (11)	−0.0225 (12)
C13B	0.0303 (11)	0.0264 (12)	0.0319 (12)	−0.0005 (9)	−0.0056 (9)	−0.0088 (9)
C14B	0.0116 (7)	0.0102 (8)	0.0180 (8)	−0.0024 (6)	0.0000 (6)	−0.0023 (6)
C15B	0.0119 (8)	0.0171 (9)	0.0162 (8)	0.0017 (7)	0.0006 (6)	0.0023 (7)
C16B	0.0170 (8)	0.0159 (9)	0.0178 (9)	0.0007 (7)	0.0006 (7)	0.0005 (7)
C17B	0.0261 (10)	0.0240 (11)	0.0158 (9)	0.0009 (8)	−0.0028 (7)	0.0017 (8)
C18B	0.0253 (10)	0.0233 (11)	0.0178 (9)	0.0051 (8)	0.0037 (7)	0.0071 (8)
C19B	0.0180 (8)	0.0164 (10)	0.0257 (10)	0.0001 (7)	0.0074 (7)	0.0020 (8)
C20B	0.0128 (8)	0.0194 (10)	0.0211 (9)	−0.0014 (7)	0.0022 (7)	0.0005 (7)

### Geometric parameters (Å, º)

**Table:**

Cl1A—C16A	1.7280 (19)	Cl1B—C16B	1.730 (2)
Cl2A—C18A	1.7290 (19)	Cl2B—C18B	1.7255 (19)
Cl3A—C19A	1.727 (2)	Cl3B—C19B	1.725 (2)
O1A—C14A	1.230 (2)	O1B—C14B	1.236 (2)
N1A—C14A	1.347 (2)	N1B—C14B	1.349 (2)
N1A—C15A	1.422 (2)	N1B—C15B	1.417 (2)
N1A—H1NA	0.78 (2)	N1B—H1NB	0.84 (2)
C1A—C6A	1.387 (3)	C1B—C2B	1.390 (3)
C1A—C2A	1.392 (3)	C1B—C6B	1.391 (3)
C1A—H1AA	0.9500	C1B—H1BA	0.9500
C2A—C3A	1.380 (3)	C2B—C3B	1.373 (4)
C2A—H2AA	0.9500	C2B—H2BA	0.9500
C3A—C4A	1.389 (3)	C3B—C4B	1.371 (4)
C3A—H3AA	0.9500	C3B—H3BA	0.9500
C4A—C5A	1.387 (3)	C4B—C5B	1.386 (3)
C4A—H4AA	0.9500	C4B—H4BA	0.9500
C5A—C6A	1.398 (3)	C5B—C6B	1.383 (3)
C5A—H5AA	0.9500	C5B—H5BA	0.9500
C6A—C7A	1.527 (2)	C6B—C7B	1.515 (3)
C7A—C8A	1.525 (2)	C7B—C14B	1.525 (2)
C7A—C14A	1.531 (2)	C7B—C8B	1.530 (2)
C7A—H7AA	1.0000	C7B—H7BA	1.0000
C8A—C13A	1.392 (3)	C8B—C13B	1.389 (3)
C8A—C9A	1.397 (3)	C8B—C9B	1.391 (3)
C9A—C10A	1.395 (3)	C9B—C10B	1.394 (3)
C9A—H9AA	0.9500	C9B—H9BA	0.9500
C10A—C11A	1.383 (3)	C10B—C11B	1.377 (4)
C10A—H10A	0.9500	C10B—H10B	0.9500
C11A—C12A	1.376 (3)	C11B—C12B	1.385 (4)
C11A—H11A	0.9500	C11B—H11B	0.9500
C12A—C13A	1.394 (3)	C12B—C13B	1.385 (3)
C12A—H12A	0.9500	C12B—H12B	0.9500
C13A—H13A	0.9500	C13B—H13B	0.9500
C15A—C20A	1.393 (3)	C15B—C20B	1.389 (3)
C15A—C16A	1.396 (2)	C15B—C16B	1.391 (2)
C16A—C17A	1.389 (3)	C16B—C17B	1.387 (3)
C17A—C18A	1.382 (3)	C17B—C18B	1.384 (3)
C17A—H17A	0.9500	C17B—H17B	0.9500
C18A—C19A	1.392 (3)	C18B—C19B	1.389 (3)
C19A—C20A	1.392 (3)	C19B—C20B	1.386 (3)
C20A—H20A	0.9500	C20B—H20B	0.9500
			
C14A—N1A—C15A	122.86 (14)	C14B—N1B—C15B	121.26 (15)
C14A—N1A—H1NA	121.7 (17)	C14B—N1B—H1NB	118.6 (15)
C15A—N1A—H1NA	113.9 (17)	C15B—N1B—H1NB	117.9 (15)
C6A—C1A—C2A	120.89 (18)	C2B—C1B—C6B	120.5 (2)
C6A—C1A—H1AA	119.6	C2B—C1B—H1BA	119.7
C2A—C1A—H1AA	119.6	C6B—C1B—H1BA	119.7
C3A—C2A—C1A	120.80 (19)	C3B—C2B—C1B	119.9 (2)
C3A—C2A—H2AA	119.6	C3B—C2B—H2BA	120.0
C1A—C2A—H2AA	119.6	C1B—C2B—H2BA	120.0
C2A—C3A—C4A	118.83 (19)	C4B—C3B—C2B	120.3 (2)
C2A—C3A—H3AA	120.6	C4B—C3B—H3BA	119.9
C4A—C3A—H3AA	120.6	C2B—C3B—H3BA	119.9
C5A—C4A—C3A	120.55 (19)	C3B—C4B—C5B	119.8 (2)
C5A—C4A—H4AA	119.7	C3B—C4B—H4BA	120.1
C3A—C4A—H4AA	119.7	C5B—C4B—H4BA	120.1
C4A—C5A—C6A	120.85 (18)	C6B—C5B—C4B	121.1 (2)
C4A—C5A—H5AA	119.6	C6B—C5B—H5BA	119.4
C6A—C5A—H5AA	119.6	C4B—C5B—H5BA	119.4
C1A—C6A—C5A	118.08 (18)	C5B—C6B—C1B	118.3 (2)
C1A—C6A—C7A	123.08 (16)	C5B—C6B—C7B	118.50 (18)
C5A—C6A—C7A	118.76 (16)	C1B—C6B—C7B	123.21 (17)
C8A—C7A—C6A	113.98 (14)	C6B—C7B—C14B	111.80 (15)
C8A—C7A—C14A	107.78 (14)	C6B—C7B—C8B	114.76 (15)
C6A—C7A—C14A	112.10 (14)	C14B—C7B—C8B	108.43 (14)
C8A—C7A—H7AA	107.6	C6B—C7B—H7BA	107.2
C6A—C7A—H7AA	107.6	C14B—C7B—H7BA	107.2
C14A—C7A—H7AA	107.6	C8B—C7B—H7BA	107.2
C13A—C8A—C9A	118.95 (18)	C13B—C8B—C9B	119.05 (19)
C13A—C8A—C7A	119.73 (16)	C13B—C8B—C7B	118.58 (17)
C9A—C8A—C7A	121.23 (16)	C9B—C8B—C7B	122.36 (18)
C10A—C9A—C8A	119.9 (2)	C8B—C9B—C10B	119.9 (2)
C10A—C9A—H9AA	120.0	C8B—C9B—H9BA	120.1
C8A—C9A—H9AA	120.0	C10B—C9B—H9BA	120.1
C11A—C10A—C9A	120.4 (2)	C11B—C10B—C9B	120.7 (2)
C11A—C10A—H10A	119.8	C11B—C10B—H10B	119.7
C9A—C10A—H10A	119.8	C9B—C10B—H10B	119.7
C12A—C11A—C10A	120.1 (2)	C10B—C11B—C12B	119.6 (2)
C12A—C11A—H11A	120.0	C10B—C11B—H11B	120.2
C10A—C11A—H11A	120.0	C12B—C11B—H11B	120.2
C11A—C12A—C13A	120.1 (2)	C13B—C12B—C11B	120.1 (2)
C11A—C12A—H12A	120.0	C13B—C12B—H12B	120.0
C13A—C12A—H12A	120.0	C11B—C12B—H12B	120.0
C8A—C13A—C12A	120.6 (2)	C12B—C13B—C8B	120.7 (2)
C8A—C13A—H13A	119.7	C12B—C13B—H13B	119.6
C12A—C13A—H13A	119.7	C8B—C13B—H13B	119.6
O1A—C14A—N1A	122.55 (16)	O1B—C14B—N1B	122.29 (16)
O1A—C14A—C7A	121.42 (15)	O1B—C14B—C7B	122.34 (15)
N1A—C14A—C7A	116.03 (14)	N1B—C14B—C7B	115.37 (14)
C20A—C15A—C16A	118.39 (16)	C20B—C15B—C16B	119.08 (17)
C20A—C15A—N1A	118.89 (16)	C20B—C15B—N1B	118.45 (16)
C16A—C15A—N1A	122.68 (16)	C16B—C15B—N1B	122.46 (17)
C17A—C16A—C15A	121.05 (17)	C17B—C16B—C15B	120.31 (18)
C17A—C16A—Cl1A	117.62 (14)	C17B—C16B—Cl1B	119.22 (15)
C15A—C16A—Cl1A	121.32 (14)	C15B—C16B—Cl1B	120.47 (14)
C18A—C17A—C16A	119.91 (18)	C18B—C17B—C16B	120.06 (18)
C18A—C17A—H17A	120.0	C18B—C17B—H17B	120.0
C16A—C17A—H17A	120.0	C16B—C17B—H17B	120.0
C17A—C18A—C19A	119.95 (17)	C17B—C18B—C19B	120.09 (17)
C17A—C18A—Cl2A	118.41 (15)	C17B—C18B—Cl2B	118.85 (15)
C19A—C18A—Cl2A	121.63 (15)	C19B—C18B—Cl2B	121.06 (16)
C20A—C19A—C18A	119.87 (18)	C20B—C19B—C18B	119.56 (18)
C20A—C19A—Cl3A	118.87 (15)	C20B—C19B—Cl3B	118.63 (15)
C18A—C19A—Cl3A	121.26 (15)	C18B—C19B—Cl3B	121.79 (15)
C19A—C20A—C15A	120.80 (17)	C19B—C20B—C15B	120.78 (18)
C19A—C20A—H20A	119.6	C19B—C20B—H20B	119.6
C15A—C20A—H20A	119.6	C15B—C20B—H20B	119.6
			
C6A—C1A—C2A—C3A	−0.4 (3)	C6B—C1B—C2B—C3B	−0.2 (3)
C1A—C2A—C3A—C4A	−0.2 (3)	C1B—C2B—C3B—C4B	−0.4 (3)
C2A—C3A—C4A—C5A	0.5 (3)	C2B—C3B—C4B—C5B	0.6 (4)
C3A—C4A—C5A—C6A	−0.1 (3)	C3B—C4B—C5B—C6B	−0.2 (4)
C2A—C1A—C6A—C5A	0.8 (3)	C4B—C5B—C6B—C1B	−0.4 (3)
C2A—C1A—C6A—C7A	177.36 (17)	C4B—C5B—C6B—C7B	178.9 (2)
C4A—C5A—C6A—C1A	−0.5 (3)	C2B—C1B—C6B—C5B	0.6 (3)
C4A—C5A—C6A—C7A	−177.25 (17)	C2B—C1B—C6B—C7B	−178.70 (18)
C1A—C6A—C7A—C8A	134.20 (17)	C5B—C6B—C7B—C14B	150.94 (17)
C5A—C6A—C7A—C8A	−49.2 (2)	C1B—C6B—C7B—C14B	−29.8 (2)
C1A—C6A—C7A—C14A	11.4 (2)	C5B—C6B—C7B—C8B	−85.0 (2)
C5A—C6A—C7A—C14A	−172.01 (16)	C1B—C6B—C7B—C8B	94.3 (2)
C6A—C7A—C8A—C13A	134.90 (17)	C6B—C7B—C8B—C13B	172.77 (17)
C14A—C7A—C8A—C13A	−99.99 (19)	C14B—C7B—C8B—C13B	−61.4 (2)
C6A—C7A—C8A—C9A	−48.6 (2)	C6B—C7B—C8B—C9B	−8.2 (2)
C14A—C7A—C8A—C9A	76.5 (2)	C14B—C7B—C8B—C9B	117.61 (18)
C13A—C8A—C9A—C10A	1.4 (3)	C13B—C8B—C9B—C10B	−1.3 (3)
C7A—C8A—C9A—C10A	−175.14 (17)	C7B—C8B—C9B—C10B	179.69 (17)
C8A—C9A—C10A—C11A	−1.0 (3)	C8B—C9B—C10B—C11B	0.8 (3)
C9A—C10A—C11A—C12A	−0.5 (3)	C9B—C10B—C11B—C12B	0.3 (3)
C10A—C11A—C12A—C13A	1.4 (3)	C10B—C11B—C12B—C13B	−0.9 (3)
C9A—C8A—C13A—C12A	−0.4 (3)	C11B—C12B—C13B—C8B	0.4 (3)
C7A—C8A—C13A—C12A	176.15 (18)	C9B—C8B—C13B—C12B	0.7 (3)
C11A—C12A—C13A—C8A	−1.0 (3)	C7B—C8B—C13B—C12B	179.76 (18)
C15A—N1A—C14A—O1A	3.2 (3)	C15B—N1B—C14B—O1B	6.1 (3)
C15A—N1A—C14A—C7A	−175.56 (16)	C15B—N1B—C14B—C7B	−173.02 (16)
C8A—C7A—C14A—O1A	−53.7 (2)	C6B—C7B—C14B—O1B	77.7 (2)
C6A—C7A—C14A—O1A	72.6 (2)	C8B—C7B—C14B—O1B	−49.8 (2)
C8A—C7A—C14A—N1A	125.16 (16)	C6B—C7B—C14B—N1B	−103.22 (18)
C6A—C7A—C14A—N1A	−108.62 (17)	C8B—C7B—C14B—N1B	129.28 (16)
C14A—N1A—C15A—C20A	113.08 (19)	C14B—N1B—C15B—C20B	110.5 (2)
C14A—N1A—C15A—C16A	−69.2 (2)	C14B—N1B—C15B—C16B	−68.5 (2)
C20A—C15A—C16A—C17A	0.6 (3)	C20B—C15B—C16B—C17B	−3.6 (3)
N1A—C15A—C16A—C17A	−177.11 (16)	N1B—C15B—C16B—C17B	175.38 (17)
C20A—C15A—C16A—Cl1A	179.03 (13)	C20B—C15B—C16B—Cl1B	176.16 (14)
N1A—C15A—C16A—Cl1A	1.3 (2)	N1B—C15B—C16B—Cl1B	−4.8 (2)
C15A—C16A—C17A—C18A	0.9 (3)	C15B—C16B—C17B—C18B	1.2 (3)
Cl1A—C16A—C17A—C18A	−177.56 (14)	Cl1B—C16B—C17B—C18B	−178.54 (15)
C16A—C17A—C18A—C19A	−2.0 (3)	C16B—C17B—C18B—C19B	2.1 (3)
C16A—C17A—C18A—Cl2A	176.97 (14)	C16B—C17B—C18B—Cl2B	−176.94 (15)
C17A—C18A—C19A—C20A	1.5 (3)	C17B—C18B—C19B—C20B	−3.1 (3)
Cl2A—C18A—C19A—C20A	−177.41 (14)	Cl2B—C18B—C19B—C20B	175.99 (14)
C17A—C18A—C19A—Cl3A	−179.15 (14)	C17B—C18B—C19B—Cl3B	178.72 (15)
Cl2A—C18A—C19A—Cl3A	2.0 (2)	Cl2B—C18B—C19B—Cl3B	−2.2 (2)
C18A—C19A—C20A—C15A	0.1 (3)	C18B—C19B—C20B—C15B	0.6 (3)
Cl3A—C19A—C20A—C15A	−179.32 (13)	Cl3B—C19B—C20B—C15B	178.91 (14)
C16A—C15A—C20A—C19A	−1.1 (3)	C16B—C15B—C20B—C19B	2.7 (3)
N1A—C15A—C20A—C19A	176.72 (16)	N1B—C15B—C20B—C19B	−176.36 (16)

### Hydrogen-bond geometry (Å, º)

**Table:**

*D*—H···*A*	*D*—H	H···*A*	*D*···*A*	*D*—H···*A*
N1*A*—H1*NA*···O1*B*^i^	0.78 (2)	2.09 (2)	2.8379 (19)	161 (2)
N1*B*—H1*NB*···O1*A*	0.84 (2)	1.94 (2)	2.7684 (19)	168 (2)
C7*A*—H7*AA*···O1*B*^i^	1.00	2.33	3.234 (2)	151
C1*B*—H1*BA*···O1*B*	0.95	2.48	3.116 (3)	125
C3*B*—H3*BA*···O1*B*^ii^	0.95	2.42	3.368 (3)	172
C12*B*—H12*B*···Cl2*B*^iii^	0.95	2.82	3.641 (3)	145
C7*B*—H7*BA*···O1*A*	1.00	2.46	3.341 (2)	147

Symmetry codes: (i) *x*+1/2, *y*, −*z*+1/2; (ii) −*x*, *y*−1/2, −*z*+1/2; (iii) *x*, −*y*+5/2, *z*+1/2.
